# Sex contribution to average age at onset of Huntington’s disease depends on the number of (CAG)_n_ repeats

**DOI:** 10.1038/s41598-024-64105-5

**Published:** 2024-07-08

**Authors:** Anna Stanisławska-Sachadyn, Michał Krzemiński, Daniel Zielonka, Magdalena Krygier, Ewa Ziętkiewicz, Jarosław Sławek, Janusz Limon, REGISTRY investigators of the European Huntington’s Disease Network (EHDN)

**Affiliations:** 1 Department of Biotechnology and Microbiology, Gdańsk University of Technology, 80-233 Gdańsk, Poland; 2 Department of Biology and Medical Genetics, Medical University of Gdańsk, 80-211 Gdańsk, Poland; 3 BioTechMed Center, Gdańsk University of Technology, Narutowicza 11/12, 80-233 Gdańsk, Poland; 4 Institute of Applied Mathematics , Gdańsk University of Technology, 80-233 Gdańsk, Poland; 5 Department of Public Health, Poznań University of Medical Sciences, 60-812 Poznan, Poland; 6 Department of Developmental Neurology, Medical University of Gdansk, 80-952 Gdańsk, Poland; 7 Institute of Human Genetics, Polish Academy of Sciences, 60-479 Poznan, Poland; 8 Department of Neurology, St. Adalbert Hospital, Copernicus PL, 80-462, Gdańsk, Poland; 9 Department of Neurological and Psychiatric Nursing, Faculty of Health Sciences, Medical University of Gdańsk, 80-211 Gdańsk, Poland; 10 Department of Medical Ethics, Medical University of Gdańsk, 80-211 Gdańsk, Poland

**Keywords:** Medical genetics, Huntington's disease

## Abstract

Huntington’s disease (HD) is a hereditary neurodegenerative disorder caused by the extension of the CAG repeats in exon 1 of the *HTT* gene and is transmitted in a dominant manner. The present study aimed to assess whether patients’ sex, in the context of mutated and normal allele length, contributes to age on onset (AO) of HD. The study population comprised a large cohort of 3723 HD patients from the European Huntington’s Disease Network’s REGISTRY database collected at 160 sites across 17 European countries and in one location outside Europe. The data were analyzed using regression models and factorial analysis of variance (ANOVA) considering both mutated allele length and sex as predictors of patients’ AO. AO, as described by the rater’s estimate, was found to be later in affected women than in men across the whole population. This difference was most pronounced in a subgroup of 1273 patients with relatively short variants of the mutated allele (40–45 CAG repeats) and normal alleles in a higher half of length distribution—namely, more than 17 CAG repeats; however, it was also observed in each group. Our results presented in this observational study point to sex-related differences in AO, most pronounced in the presence of the short mutated and long normal allele, which may add to understanding the dynamics of AO in Huntington’s Disease.

**Trial registration**: *ClinicalTrials.gov identifier* NCT01590589.

## Introduction

Huntington’s disease (HD) is an autosomal dominant, progressive, neurodegenerative disorder characterized by motor symptoms, cognitive impairment, and psychiatric disturbances. The prevalence of HD is 6.37 and 8.87 per 100,000 people in Europe and North America, respectively, while it is much less prevalent in Africa and Asia^1^. It is caused by an expansion of the trinucleotide CAG tandem repeat (> 35 CAGs) located in exon 1 of the *HTT* gene encoding the huntingtin protein. Huntingtin’s cellular functions are not fully understood, although mutant huntingtin is known to be digested into fragments, which build toxic aggregates that disrupt transcription processes and cytoplasmic transport^2^. This leads to mitochondrial dysfunction, altered reactive oxygen species defense, and finally, apoptosis resulting in neuronal dysfunction.

The onset of the disease usually occurs in midlife. Age at onset (AO) is strongly associated with the length of the (CAG)_n_ expansion in the mutated allele, such that the longer the expanded repeat is, the earlier the onset of clinical symptoms will manifest. Mutated alleles that carry at least 40 repeats are fully penetrant^3^. Expanded alleles ranging from 40 to 45 repeats are observed most often.

A considerable variance in AO has been observed in individuals who carry the (CAG)_n_ expansion at comparable lengths, prompting the search for modifying factors that may account for the varying AO. The length of CAG repeats is responsible for approximately 50–70% of the variability of AO in HD^2^.

The mutated *HTT* is highly unstable, and both extensions and contractions of the mutated allele in inter-generational transmissions are observed in HD^4^. Interestingly, the sex of a mouse embryo has an impact on the likelihood of CAG repeat contractions or expansions, which has led to suggestions that the change in CAG repeat number may also be a post-zygotic event^5,6^. In humans, it has been observed that the mutated allele may contract when inherited from mother^7,8^. Notably, 42% of maternally transmitted alleles contracted when passed to daughters and only 27% when passed to sons^9^. The above observation led to a suggestion that both parental and offspring sex plays a role in HD pathology. Interestingly, data regarding the impact of sex on AO of HD are relatively scarce^10–13^, and it is clear that the involvement of the patient’s sex in the AO of HD should be further evaluated in the context of the size of the mutated allele.

In recent years it has been noticed that the expansion of CAG triplets occurs not only in germinal but also in somatic cells, including the nervous system—specifically, in human striatal cells—and is an early event in the disease course^14^. CAG trinucleotide instability has been observed in non-dividing cells^15,16^, it has been demonstrated that the expansions of a mutated allele in the cortex^17^ and in the post-mitotic striatum^16^ determine the AO of HD.

The mechanism by which expansions arise may be a consequence of the physiological processes present in somatic cells^17^. It has been recently established that more genetic factors are involved in the mechanism by which the expansion in non-dividing cells arises. Somatic instability is dependent on the presence of mismatch repair proteins—namely Msh2^18^, Msh3 and Mlh1^19^, Msh2-Msh3 form MutS-beta heterodimer recognizing and binding CAG loops. In the genome-wide association study (GWAS), other DNA repair system genes—the *FAN1* and the *RRM2B*—have been identified as genetic factors that underlie variation in AO^20^. The *FAN1* gene encodes a nuclease involved in inter-strand DNA crosslink repair that is necessary for controlling CAG repeat expansion^21^. Another GWAS study confirmed that the *MLH1* gene is a factor modifying AO in HD^22^. It has been further hypothesized that the mismatch repair system (MMR) may introduce expansion while correcting for misalign of DNA strands during its reannealing, either in the course of DNA replication in dividing cells or RNA transcription in non-dividing cells, in a process initialized by binding of PCNA and MutS-beta^23^.

Apart from the mutated allele, the role of a normal allele in HD pathogenesis has been also considered. The negative correlation between AO and the normal CAG repeat number has been reported to be sex-specific and stronger for normal maternal allele transmissions^24^. Irrespective of the patient’s sex a negative correlation between AO and the normal paternal allele size has also been reported^25^. Interestingly, results of analyses of 337 inter-generational transmissions indicate that, in patients who inherit the expanded allele from their mothers, the increased frequency of mutated allele contractions has been associated with longer normal alleles^26^, pointing at differences in the interplay between mutated and normal alleles between sexes in trans-generational length changes. Next, larger normal alleles in combination with shorter mutated alleles were associated with earlier AO and vice versa*—*in combination with longer mutated alleles—with later AO, in a group of 921 subjects^27^. Similarly, the longest normal alleles were linked to later AO when combined with long expanded alleles^28^. Nevertheless, no impact of the normal allele length on motor onset along the mutated allele length was found in multiple linear regression analyses in a large population of 4067 subjects^29^.

The present understanding of the impact of a variety of factors on AO of HD remains not fully solved. The present study aimed to test the extent to which the sex of affected individuals can impact AO of HD in the context of the length of CAG repeats within large and normal alleles. The analyses were conducted in a large-scale, multi-national cohort of European ancestry. This observational study supported by statistical analyses adds to understanding the pathogenesis of HD in a way that could not be achieved in animal or cellular models.

## Methods

### Study population

Analyses were performed on data extracted from the REGISTRY database provided by the European Huntington’s Disease Network (EHDN). The data were obtained as part of the EDHN’s data mining project 0636. REGISTRY data were collected at 160 sites across in 18 countries (17 European and one country outside Europe) from June 2004 and were assessed in November 2017. Given that the data required to perform the analyses were not available for all the HD patients included in the REGISTRY database, the selection of the study population was necessary. The criteria for including subjects were (i) the number of (CAG)_n_ repeats in the expanded allele equal to or higher than 36 (10,363 subjects) and (ii) the availability of the clinician’s best estimate of AO—referred to as the rater’s estimate that reflects most probable onset age based on experienced professional interview with patient and family members or onset established examining person observed from premanifest HD stage, the number of (CAG)_n_ repeats in the normal allele, if available, lower than 36 (3723 subjects). The rater’s estimate of AO was calculated based on the “sxrater,” which is coded as a date in the REGISTRY database. The EHDN investigators’ AO estimation was used in analyzes. The data on the (CAG)_n_ allele length were from the EHDN database or, if not available, from the local laboratory. The number of repeated CAG units in the expanded allele ranged from 36 to 90. AO was analyzed in the context of patients’ sex in a group of 3723 patients (see Table 1 for further details).

Ethical approval for REGISTRY was obtained in each participating country. All participants gave written informed consent: https://www.enroll-hd.org/enrollhd_documents/2016-10-R1/registry-protocol-3.0.pdf. The REGISTRY protocol was approved by the EHDN Scientific and Bioethics Advisory Committee.

### Statistical analyses

Multiple regression models to estimate the variance in patients’ AO were created. The regression coefficients from the analyzed models were used to assess the proportion of variance in patients’ AO (the outcome measure) explained by mutated allele length and the other predictor variable: the sex of HD patients or a type of first symptoms. Regression coefficients were calculated while the expanded allele sizes were median-centered for 43 CAG repeats. The interaction terms between the mutated allele length and patients’ sex were not significant in any of the models (Table 2, models A–F); therefore, models without interaction terms were chosen. In multiple linear regression models designed to determine whether the impact of a first symptom type differed between sexes, the significance of the interaction coefficients term between the patient’s sex and type of first symptoms were of interest.

In simple linear regression analyses, the coefficient of determination (R^2^) was used to assess the proportion of variance in AO explained by the predictor variable.

A two-way ANOVA using a generalized linear model with the LSMEAN statement and Tukey post-hoc test was performed. The association between AO and the number of (CAG)_n_ repeats in the mutated allele with the impact of patients’ sex was assessed using factorial analyses with a two between-subjects factor.

In both the factorial and regression analyses, AO, which was a dependent variable, was natural log-transformed^30^. The normality of the distributions was assessed using the Kolmogorov–Smirnov test.

In the ranked analyses, the significance of differences between the groups was determined using the Wilcoxon rank–sum test, corrected for multiple comparisons using the Bonferroni method when necessary. Chi-square statistics were used to test the difference in distribution of the categorical variables (e.g., frequency of first symptom types between sexes). Correlations between the number of CAG repeats within either normal or mutated allele, and the AO values were calculated using Spearman statistics.

The level of significance was set at 0.05. The statistical analyses were carried out using SAS versions 9.3 and 9.4 (NC, USA) and the R platform.

### Population stratifications

Several analyses were performed in subgroups defined according to the ranges of the expanded allele size: ≤ 39 (CAG)_n_, 40–45 (CAG)_n_, 46–50 (CAG)_n_, and > 50 (CAG)_n_. Subjects in group 1 had alleles of 39 CAG repeats or fewer, which are not fully penetrant^3^. Carriers of penetrant and mid-size mutation of 40–50 CAGs had an average age at onset HD. They are the majority of HD patients whose disease is characterized by a large diversity in AO; thus, it was further divided into group 2, including subjects who had expanded alleles of 40–45 CAGs, and group 3 who had expanded alleles of 46–49 CAGs. Subjects in group 4 carried mutation longer than 50 CAGs and had juvenile form of HD.

Expanded alleles described 27.85% of the variance in AO among 2617 carriers of 40–45 CAGs (70.25% of those for whom sex, AO, and mutated allele length were available), 10.68% among 757 carriers of 46–50 CAGs, and 60.04% among 255 carriers of alleles longer than 50 CAGs, who established an early-onset subgroup. Thus, the coefficient of determination (R2) in regression models, which is used to assess the proportion of variance in AO explained by the expanded allele size in the whole cohort is highly influenced by the characteristics of early-onset subjects, for whom genetic factor plays the strongest role in HD pathogenesis. Thus, the precision in determining the influence of an additional factor on AO, specifically of the patient’s sex, increases in analyses conducted among subgroups of mutated allele sizes, which certainly have a dominant impact.

Moreover, in several analyses, the population has been stratified into two groups regarding the size of a normal allele, that is, below/equal to and above a median value of 17 CAGs. Subjects in a group of lower normal allele length distribution carried 8–17 CAG repeats, while subjects in a group of higher normal allele length distribution carried 18–35 CAG repeats.

## Results

### Data distribution and basic statistics

A clinician’s best estimate of AO (i.e., rater’s estimated AO) and expanded allele length were available for 3723 subjects: men accounted for 47.95% and women for 52.05% (Table 1). Data pertaining to normal alleles were available for 3643 subjects. The frequencies of the first symptom types, as listed in rater’s estimate, were comparable between women and men (P = 0.2294, Table 1), although a slightly higher proportion of men first observed motor (52.82%) and cognitive symptoms (7.90%) compared to women (51.48% and 6.71%, respectively), while more women first observed mixed symptoms (19.76%) than men did (17.95%). These differences between sexes were non-significant, whether assessed using chi-square statistics (Table 1) or multiple regression analyses, which showed that the interaction term between the patient’s sex and the first symptom types did not contribute significantly to explaining the proportion of variance in AO (data not presented).

**Table 1:** Study population characteristics, with regards to HD patients’ sex; for the whole study group and among those for whom both alleles within the *HTT* gene were known.

	All	Men	Women	*P*
				Males vs females
^1^Study group	N = 3723	N = 1785 (47.95%)	N = 1938 (52.05%)	
Age at onset (rater’s estimate)				
Median (q1–q3) Mean, SD		44 (35–52) 43.96, 12.29	45 (36–53) 44.61, 12.30	0.0793^#^
First symptoms				
1 (motor)		N = 936 (52.82%)	N = 990 (51.48%)	0.2294*
2 (cognitive)		N = 140 (7.90%)	N = 129 (6.71%)	
3 (psychiatric)		N = 358 (20.20%)	N = 402 (20.90%)	
4 (oculomotor)		–	N = 3 (0.16%)	
5 (other)		N = 20 (1.13%)	N = 19 (0.99%)	
6 (mixed)		N = 319 (17.95%)	N = 380 (19.76%)	
Expanded allele length				
Median (q1–q3) Mean, SD		43 (42–46) 44.31, 4.26	43 (42–46) 44.41, 4.41	0.4376^#^
^2^Subgroup with data on normal allele length	N = 3643	N = 1749	N = 1894	
Normal allele length				
Median (q1–q3) Mean, SD		18 (17–20) 18.49, 3.19	17 (17–19) 18.41, 3.28	0.1431^#^

^1^Data restricted to subjects whose expanded allele ≥ CAG_36_, normal allele < CAG_36_ (if available), for whom sex, AO, number of (CAG)_n_ was available. Homozygous subjects (genotype: 42/37, 44/36, 44/39, 45/45, 49/39, 54/37, 54/37, 65/36) were excluded.

^2^Data restricted to subjects for whom numbers of (CAG)_n_ within both alleles were available.

^#^Wilcoxon Rank Sum Test.

*Chi-square.

A simple group comparison revealed no statistically significant difference in AO between men and women (Table 1). The median AO was 44 years for men and 45 for women (*P* = 0.0793), while the median length of the expanded allele was 43 for both men and women (*P* = 0.4376).

As expected, the mutation size correlated highly with AO both in women and men (r = − 0.75863, r = − 0.76530, respectively; both *P* < 0.0001). In the study population analyzed as a whole, the size of a mutated allele described 60.65% of the variance in AO—61.30% in women and 60.18% in men.

### Association between patients’ sex and age at onset in the context of mutated allele length

In the whole study population, in regression models describing the proportion of variance in AO and involving the mutated allele length and patient sex as two predictor variables, sex was significantly involved in the AO variability (*P* = 0.0012; Table 2A). After the cohort was divided with respect to mutation size, this association stayed significant among those subjects who had 40–45 CAG repeats (*P* = 0.0006; Table 3B) but not in those who had ≤ 39 (CAG)_n_, 46–50 (CAG)_n_ and > 50 (CAG)_n_ (data not presented).

**Table 2:** Multiple linear regression models describing the proportion of variance in AO explained by mutated allele and sex of HD patient, either with or without interaction between mutation and patient's sex.

Intercept (P-value)^1^	^1^Mutated allele (P-value)^1^	^2^Sex of HD patient (P-value)	Interaction between mutation and patient’s sex (P-value)	R^2^ (P-value)^1^	N
(A) Whole population					
3.81443	− 0.05896	0.01852 (0.0076)	0.00222 (0.1464)	0.60781	3723
3.81288	− 0.05776	0.02152 (0.0012)	–	0.60759	
(B) Patients with mutated allele of 40–45 CAG					
3.81690	− 0.07993	0.02734 (0.0003)	0.00510 (0.2935)	0.28211	2617
3.81802	− 0.07732	0.02526 (0.0006)	–	0.28180	
(C) Patients with normal allele > 17 CAG					
3.80836	− 0.05743	0.02937 (0.0045)	0.00263 (0.2352)	0.59334	1802
3.80635	− 0.05589	0.03312 (0.0007)	–	0.59302	
(D) Patients with normal allele ≤ 17 CAG					
3.81982	− 0.06022	0.01041 (0.2738)	− 0.00014 (0.9468)	0.62755	1841
3.81991	− 0.06028	0.01023 (0.2615)	–	0.62755	
(E) Patients with normal allele > 17 CAG, with mutated allele of 40–45 CAG					
3.80952	− 0.07574	0.04584 (< 0.0001)	0.00653 (0.3746)	0.24131	1273
3.81080	− 0.07252	0.04340 (< 0.0001)	–	0.24084	
(F) Patients with normal allele ≤ 17 CAG, with mutated allele of 40–45 CAG					
3.82523	− 0.08362	0.01027 (0.3209)	0.00180 (0.7843)	0.33226	1285
3.82564	− 0.08269	0.00952 (0.3398)	–	0.33222	

Independent variables were median-centered, thus an intercept equals ln(AO) while mutated allele equals 43.

^1^P < 0.0001.

^2^Males were reference sex.

**Figure 1:**
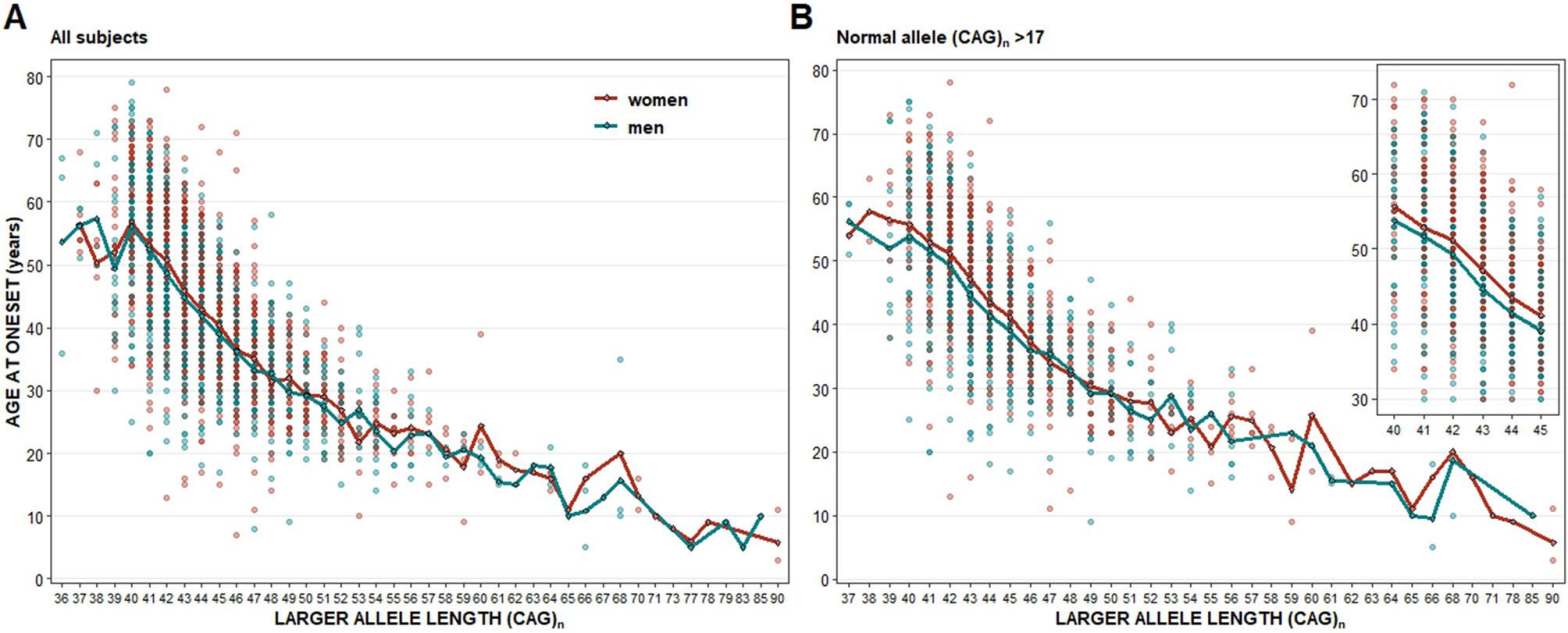
Age at onset of male and female patients in relation to the number of CAG repeats within the mutated allele (**A**) across the entire study group (**B**) among subjects who carry more than 17 CAG repeats within the normal allele (higher half of the length distribution), inset: analyses for subjects with mutated allele (CAG)_n_ = 40–45. Two-way ANOVA and regression analyses were performed with natural log-transformed AO.

Similarly, in two-way ANOVA LSMEAN analyzes, patients’ sex had a significant impact on AO variability across the entire study population (*P* = 0.0004; Table 3A, Fig. 1 A). In those analyzes, unlike in the regression models, the mean AO is assessed by ANOVA between women and men for each mutated allele of the same size. Since no interaction between the length of a mutated allele and patients’ sex was found, analysis of type II was chosen^31^. In analyses performed in subgroups defined by the range of mutated allele length, patient sex had a significant impact on AO variance (*P* = 0.0005; Table 3B) among those subjects who had 40–45 CAG repeats.

The subgroup of HD patients who had expanded allele of 40–45 CAG repeats was characterized by high variance in AO, ranging from the age of 5 to 83; the median AO was 48 years (mean ± SD; 48.68 ± 9.99). Analyzes in ranges to evaluate differences in AO between women and men in the context of mutated allele length are presented in Supplementary Table S1.

**Table 3:** Impact of the sex and mutated allele length on the AO in patients, analyzed by two-way ANOVA.

Impact of expanded allele and sex on age at onset in HD						
Factor	DF	Type II SS	Mean square	F value	*P-*value	N
(A) Whole population						
Mutated allele	41	244.2525553	5.9573794	155.63	< 0.0001	3723
Sex	1	0.4797997	0.4797997	12.53	0.0004	
Mutated allele*sex interaction	32	1.3539487	0.0423109	1.11	0.3131	
(B) Mutated allele of (CAG)_n_ = 40–45						
Mutated allele	5	35.81407630	7.16281526	204.28	< 0.0001	2617
Sex	1	0.42192903	0.42192903	12.03	0.0005	
Mutated allele*sex interaction	5	0.22615282	0.04523056	1.29	0.2653	
(C) Normal allele of (CAG)_n_ > 17						
Mutated allele	36	119.6907953	3.3247443	81.97	< 0.0001	1802
Sex	1	0.5230997	0.5230997	12.90	0.0003	
Mutated allele*sex interaction	25	1.2288383	0.0491535	1.21	0.2157	
(D) Normal allele of (CAG)_n_ ≤ 17						
Mutated allele	36	122.7888247	3.4108007	95.85	< 0.0001	1841
Sex	1	0.0729239	0.0729239	2.05	0.1525	
Mutated allele*sex interaction	24	1.3786835	0.0574451	1.61	0.0303	
(E) Normal allele of (CAG)_n_ > 17 and mutated allele of (CAG)_n_ = 40–45						
Mutated allele	5	14.95028510	2.99005702	77.82	< 0.0001	1273
Sex	1	0.59534683	0.59534683	15.50	< 0.0001	
Mutated allele*sex interaction	5	0.05386317	0.01077263	0.28	0.9240	
(F) Normal allele of (CAG)_n_ ≤ 17 and mutated allele of (CAG)_n_ = 40–45						
Mutated allele	5	20.42851681	4.08570336	129.15	< 0.0001	1285
Sex	1	0.03410713	0.03410713	1.08	0.2993	
Mutated allele*sex interaction	5	0.27334713	0.05466943	1.73	0.1252	

The analyses were carried (A) across the entire study group, and in the following subgrups: (B) in subjects with the mutated allele of (CAG)_n_ = 40–45 (C) in subjects with the normal allele of (CAG)_n_ > 17 (D) in subjects with the normal allele of (CAG)_n_ ≤ 17 (E) in subjects with the mutated allele of (CAG)_n_ = 40–45 and normal allele of (CAG)_n_ > 17 (F) in subjects with the mutated allele of (CAG)_n_ = 40–45 and normal allele of (CAG)_n_ ≤ 17.

### Association between patients’ sex and age at onset in the context of the length of both mutated and normal alleles

To evaluate whether the association between AO and patient sex might be impacted by the number of repeats within a normal allele, the study population has been stratified by the normal allele length ranges into the lower or higher half of distribution.

In the multiple regression models, patient sex was a significant factor among those who had a normal allele in the higher half of length distribution, that is, 18–35 CAG repeats (*P* = 0.0007; Table 2C) but not among those who had normal allele equal to or shorter than 17 CAG repeats (*P* = 0.2615; Table 2D).

When the population was stratified according to both mutated and normal allele length ranges, the results remained significant among those who had a mutated allele of 40–45 CAGs and a normal allele in the higher half of the length distribution (*P* < 0.0001; Table 2E). Interestingly, in this group, sex accounted for 0.80% of variation in AO (*P* = 0.0014), as assessed by simple regression analyses (data not presented). Given the complex nature of AO distribution in HD, this association appears to be quite meaningful for a binary factor such as patients’ sex.

Analogously, in factorial two-way ANOVA LSMEAN analyses, the difference in AO between affected women and men was significant only among those subjects whose normal allele length was in the higher half of the length distribution (Table 2C, Fig. 1B). This association was particularly pronounced in patients who also had 40–45 CAG repeats in the mutated allele and normal allele in a higher half of allele distribution (*P* < 0.0001; Table 3E, Fig. 1B, intercept).

Overall, the mean AO in women and men in the subgroup of 40–45 CAG repeats in the mutated allele and 18–35 CAG repeats in the normal allele was 49.40 ± 9.64 and 47.70 ± 10.03 years, respectively (*P* = 0.0071). The difference in mean AO between females and males varied from 0.55 years for those with the mutated allele of 40 CAG repeats—to 2.23 years for those with the mutated allele of 44 CAG repeats. Analyses in ranges to evaluate differences in AO between women and men in the context of both mutated and normal allele length are presented in Supplementary Table S2.

## Discussion

Expansion of CAG repeats is a well-documented HD-determining factor that was first identified during the 1990s. AO is negatively correlated with increasing number of repeats in the mutated allele, with the longest alleles (> 50 CAG repeats) determining a juvenile-onset disease. However, in patients with shorter mutated alleles a high variability in AO is observed. This has prompted a search for factors that may contribute to the variance in AO of HD.

We observed the impact of patients’ sex on AO of HD, in analyses that exclusively included subjects for whom the rater’s estimation of AO was known. Analysis of the entire study population revealed that patients’ sex was a significant factor in the variation in AO. However, among those who carried 40–45 CAG repeats, females’ AO was distinctly later than that of males. This finding comes as a surprise because earlier such observation has never been confirmed. The first estimation of sex involvement was made before the measurement of mutated allele size became a clinical practice^13^. No difference in AO between women and men among 2068 subjects^10^, among 151 subjects^32^ or in residual AO of diagnostic motor signs, in the study including 4793 subjects^11^, has been identified through group comparison analyses. Moreover, no difference in age of clinical HD diagnosis between sexes, among 2145 HD patients, was found when the analysis was controlled for the specially invented score, calculation of which involved the individual’s current age and repeat length^12^. It should be acknowledged that the group examined in the present study was of a relatively large size of 3723 subjects. A further advantage of our work was the use of the clinician’s (the rater’s) best estimate of AO and the type of statistical analyses that we applied. Although residual AO calculation involves CAG repeat length^11^, two-way ANOVA compares the AO of women and men for each mutated allele while multiple regression analyzes include mutated allele as a linear variable. Our initial observation made in the analyzes of the whole study population was followed by the selection of a group among whom the difference in AO reaches higher significance.

Given that the poly-Q mutation in the *HTT* is highly dynamic in brain tissue, leading to its mosaicism^14,16,17,33–35^, our results may suggest that either the enlargement of expansion size or the prevalence of enlargements may be higher in terminally differentiated male neurons; alternatively, the increase in contraction size or its prevalence might be higher in terminally differentiated female neurons. If so, such somatic sex-related differences in repeat instabilities may underlie our observation. Since the inter-generational and somatic changes might follow a similar direction, the data regarding inter-generational changes in expansion length may add to explaining the possible mechanism behind our observation. When parents’ and offspring’s sexes were considered in inter-generational analyses, mother–to–daughter contractions were reported^7,9^. Moreover, in mice sired by the same fathers, sex-related differences in expansion stability have been reported since expansions of the mutated allele were more frequent in males, while contractions were more frequent in females^5^.

Also, several genetic variants have been found to affect AO of HD in a sex-related manner. Recently, variants within the *MSH3*/*DHFR* locus—a proven source of the dynamic mutation instability in finally differentiated brain tissue^19^—have been found to be stronger modifiers of the diagnostic motor signs in women when analyses were set as sex-specific^11^. Moreover, X-chromosome-wide association study (XWAS) found a variant close to the moesin gene potentially modifying AO^36^. Differences in AO of HD between genders have previously been reported for carriers of different genotypes within the *APOE* gene^24^. We may speculate that those or other variants might be associated with sex-specific differences in neuronal mosaicism and thus disease AO.

A neuroprotective effect of 17ß-estradiol has been reported in rats carrying mutations of 51 CAG repeats^37^. It has been demonstrated that in neuroblastoma cells estrogen enhances the expression of huntingtin and neuroglobin, which are then complexed and bind to mitochondria in response to H_2_O_2_ stress to prevent apoptosis; these processes are abandoned in the case of mutated huntingtin^38^. Moreover, higher mtDNA levels have been observed in leukocytes from women with HD compared to men with HD^39^, which may be associated with a protective effect against oxidative stress in females.

Our findings suggest that different factors may be associated with AO between subjects who carry 40–45 CAG repeats and have average AO and those who carry longer mutated alleles and thus have earlier AO. It is well-established that juvenile HD and average-onset HD differ at the molecular level: neurons with intranuclear inclusions composed of huntingtin are observed more frequently in juvenile patients, while extracellular structures with the morphology of dystrophic neuritis dominate in the central nervous system of adult-onset patients^40^.

Further, for subjects who carried a normal allele in the higher half of its length distribution (i.e., more than 17 CAG repeats), the difference in AO between sexes was even more pronounced. The impact of the normal allele length on HD pathogenesis has been considered previously^24,25,27,28,41^. Interestingly, inter-generational contractions were more frequent when mothers carried long normal alleles^26^. Normal allele length has been demonstrated to impact AO in a manner dependent on the number of repeats in the mutated allele^27^, with long normal alleles reducing AO in the presence of the short mutated alleles. These data lead us to repeat the speculation that expansion occurs more frequently in male somatic cells of brain tissue while contractions occur more frequently in female cells—specifically, in the presence of the short mutated and long normal allele, leading to us observing the difference in AO between women and men in the presence of this constitutive blood genotype. This hypothesis, however, requires further investigation.

We conducted our analyses in a large study group comprising 3723 subjects from the EHDN REGISTRY database, which is a multi-center, multi-national, prospective, observational study of HD. The diversity of study population combined with the standardized data from patients’ examinations is the present study’s strength. However, the estimation of AO constitutes a weakness of our study. The rater’s confidence level was high for 69.91% of AO estimations in our study group. However, AO was estimated retrospectively, the estimations might have been based on family reports, sometimes several years after the onset, which could lead to a bias in AO. Moreover, the data on the CAG number within both the expanded and normal alleles have been determined in several centres, including the EHDN and local laboratories, which might contribute to a bias in our results.

In conclusion, we have presented analyses indicating that AO depends on the patient’s gender with regard to the sizes of both the mutated and the normal alleles. For patients who carry 40–45 CAG repeats, AO occurred later in females than in males. This association was stronger when the normal allele was in the upper range of the size distribution (that is, it was longer than 17 CAG repeats). Finally, the most pronounced difference in AO between sexes was observed among 1273 patients combining the above mentioned stratifications—in those with the mutation of 40–45 CAG repeats and normal allele longer than 17 CAG repeats.

### Supplementary Information

Supplementary Information.

Supplementary Tables.

## Data Availability

The REGISTRY data are not publicly available and are granted by the EHDN to qualified researchers given institutional assurance regarding patients’ data confidentiality. Anonymized patient data are granted from the EHDN following the standard submission procedure (https://ehdn.org/hd-clinicians-researchers/data-mining-projects-2/). Further information and data requests should be directed to Anna Stanisławska-Sachadyn (anna.stanislawska@pg.edu.pl).
