# Supplementary material for: Sex contribution to average age at onset of Huntington’s disease depends on the number of (CAG)n repeats
Source: Sci Rep. 2024 Jul 8;14:15729. doi: 10.1038/s41598-024-64105-5 (PMC11231309; doi:10.1038/s41598-024-64105-5)
Supplement: Supplementary file 2 — Supplementary Tables. [file 41598_2024_64105_MOESM2_ESM.pdf]

Sex contribution to average age at onset of Huntington's Disease depends on the number of $(CAG)_n$ repeats - – Supplementary Information

Anna Stanisławska-Sachadyn,[1,2,3*] Michał Krzemiński,[4] Daniel Zielonka,[5] Magdalena Krygier,[6] Ewa Ziętkiewicz,[7] Jarosław Sławek,[8,9] Janusz Limon,[10] on behalf of REGISTRY investigators of the European Huntington's Disease Network (EHDN)

[1] Department of Biotechnology and Microbiology, Gdańsk University of Technology, 80-233 Gdańsk, Poland

[2] Department of Biology and Medical Genetics, Medical University of Gdańsk, 80-211 Gdańsk, Poland

[3] BioTechMed Center, Gdańsk University of Technology, Narutowicza 11/12, 80-233, Gdańsk, Poland.

[4] Institute of Applied Mathematics, Gdańsk University of Technology, 80-233 Gdańsk, Poland

[5] Department of Public Health, Poznań University of Medical Sciences, 60-812 Poznań, Poland

[6] Department of Developmental Neurology, Medical University of Gdansk, 80-952 Gdańsk, Poland

[7] Institute of Human Genetics, Polish Academy of Sciences, 60-479 Poznań, Poland

[8] Department of Neurology, St. Adalbert Hospital, Copernicus PL, 80-462 Gdańsk, Poland

[9] Department of Neurological and Psychiatric Nursing, Faculty of Health Sciences, Medical University of Gdańsk, 80-211 Gdańsk, Poland

[10] Department of Medical Ethics, Medical University of Gdańsk, 80-211 Gdańsk, Poland

*Corresponding author:
Email: anna.stanislawska@pg.edu.pl

Association between patients' sex and age at onset, in the context of mutated allele length – ranked analyses

Differences between the AO values in male and female patients were evaluated in subgroups defined according to the ranges of the numbers of CAG repeats (Supplemental Table 1). Determination of whether there was any difference in mutated allele sizes between the cells of interest (specifically, between the groups defined by mutated allele ranges) allowed us to exclude the possibility that variability in the distribution of CAG length impacts AO.

AO in women who had 40–45 CAG repeats was significantly later than AO in men (mean±SD; 49.19±9.90 vs 48.19±10.07, respectively; P = 0.020), while no differences were observed in mutated allele length between women and men in the analyzed subgroups.

Association between patients' sex and age at onset in the context of both mutated and normal allele length – ranked analyses

AO between sexes was compared in subgroups defined by the size ranges of CAG repeats in both mutated and normal alleles (Supplemental Table 2).

The sex-related difference in AO in patients with the mutated allele of 40–45 CAG was significant only for those in whom the normal allele length was longer than 17 CAG repeats (mean±SD; 49.40±9.64 vs 47.70±10.03, in women and men, respectively; P = 0.0071).

Supplemental Table 1. Mean/median values of age at onset in women and men with HD and of mutated allele length in groups defined by mutated allele ranges

Mutated allele $(CAG)_n$	Female AO		Male AO			Female mutated allele		Male mutated allele		
	Median (q1-q3), N	Mean, SD	Median (q1-q3), N	Mean, SD	P-value	Median (q1-q3), N	Mean, SD	Median (q1-q3), N	Mean, SD	P-value
≤39	54 (50-63), 53	54.87, 11.30	54 (48-67), 41	56.88, 12.89	0.604	39 (38-39), 53	38.32, 0.87	39 (38-39), 41	38.46, 0.92	0.253
40-45	49 (42-56), 1349	49.14, 9.90	48 (41-55), 1268	48.19, 10.07	**0.020**	43 (41-44), 1349	42.62, 1.50	43 (41-44), 1268	42.57, 1.52	0.411
46-50	35 (30-39), 397	35.01, 7.08	34 (30-38), 360	34.20, 6.70	0.086	47 (46-48), 397	47.31, 1.31	47 (46-48), 360	47.40, 1.26	0.219
>50	24 (20-29), 139	24.20, 7.06	24 (19-29), 116	23.55, 7.21	0.564	54 (52-57), 139	55.81, 6.74	53 (52-57), 116	55.75, 6.60	0.736

Supplemental Table 2. Mean/median values of patient's AO and mutated allele length in groups defined by mutated allele ranges and normal allele ranges

Mutated allele $(CAG)_n$	Normal allele $(CAG)_n$	Female AO Median (q1-q3), N	Female AO Mean, SD	Male AO Median (q1-q3), N	Male AO Mean, SD	P-value	Female mutated allele Median (q1-q3), N	Female mutated allele Mean, SD	Male mutated allele Median (q1-q3), N	Male mutated allele Mean, SD	P-value
≤39	≤17	54 (50-58), 33	53.67, 10.64	52 (48-67), 25	56.40, 13.62	0.7592	39 (38-39), 33	38.33, 0.85	39 (38-39), 25	38.40, 1.00	0.5281
	>17	55 (50-63), 18	56.44, 12.56	58.5 (48.5-69), 16	57.63, 12.06	0.7955	38.5 (38-39), 18	38.22, 0.94	39 (38.5-39), 16	38.56, 0.81	0.2107
40-45	≤17	49 (42-56), 684	48.97, 10.13	48 (41-55), 601	48.62, 10.04	0.369	43 (41-44), 684	42.60, 1.50	43 (41-44), 601	42.56, 1.54	0.5541
	>17	49 (43-56), 634	49.40, 9.64	48 (41-54), 639	47.70, 10.03	**0.0071**	43 (42-44), 634	42.65, 1.49	43 (41-44), 639	42.60, 1.50	0.6432
46-50	≤17	35 (31-40), 195	35.45, 7.52	34 (30-38), 181	33.87, 6.41	0.0282*	47 (46-48), 195	47.21, 1.23	47 (46-48), 181	47.37, 1.19	0.1336
	>17	35 (30-39), 195	34.62, 6.68	35 (30-39), 171	34.50, 6.94	0.8088	47 (46-48), 195	47.39, 1.38	47 (46-48), 171	47.42, 1.34	0.7987
>50	≤17	24 (21-29.5), 64	24.55, 6.73	24 (18-29), 58	23.10, 6.83	0.2983	53 (51-55.5), 64	54.70, 5.28	53 (52-58), 58	56.10, 7.08	0.3274
	>17	24 (20-27), 71	23.70, 7.33	24 (19-29), 58	24.00, 7.60	0.7779	54 (52-58), 71	56.80, 7.81	53 (51-56), 58	55.40, 6.13	0.1448

*P-value was non-significant after correction for multiple comparisons.
